# Romanian patients’ access to clubfoot treatment services

**DOI:** 10.25122/jml-2021-0334

**Published:** 2022-02

**Authors:** Bianca Oana Duran, Marius Ionut Ungureanu

**Affiliations:** 1. Department of Public Health, Faculty of Political, Administrative and Communication Sciences, Babes-Bolyai University, Cluj-Napoca, Romania; 2. Center for Health Workforce Research and Policy, Faculty of Political, Administrative and Communication Sciences, Babes-Bolyai University, Cluj-Napoca, Romania

**Keywords:** healthcare access and outcomes, pediatric orthopedics, clubfoot, Ponseti method, health policies

## Abstract

The number of clubfoot new cases in Romania is on the rise. According to orthopedic research, the Ponseti method is the elective treatment for clubfeet. This paper aims to provide an overview of the current facilitators and barriers in accessing clubfoot treatment services in Romania and to assess the impact of care-related factors on patients’ well-being. Our research shows that nationally, few orthopedic surgeons are using the Ponseti method and most of them are concentrated only in Cluj-Napoca. Moreover, gynecologists, neonatologists, and family physicians were not informed about the initial diagnosis and the current treatment of clubfoot. Primary medical care was significantly postponed in some cases. Moreover, no clubfoot organizations were mentioned by the parents included in the study, and psychological support was provided neither for the parents nor for the children. Based on the results of our study, we conclude that more efforts need to be done for the diagnosis and treatment of children with clubfeet. These include actions which are focused on raising awareness around this abnormality and more well-developed treatment guidelines.

## Introduction

Clubfoot is the most common abnormality in the musculoskeletal system. Clubfoot may be associated with myelodysplasia or arthrogryposis but is most frequently an isolated birth defect and is considered idiopathic [1–2].

Globally, clubfoot affects around 174,000 children annually, 90% of whom live in low- and middle-income countries [3]. At a European level, between 2005–2009, there were 5,458 congenital clubfoot cases, of which 5,056 (93%) were live-born infants [4]. In Romania, clubfoot affects 1 in 1,000 live births per year, and according to recent research, men are twice as likely to have clubfeet, which indicates a genetic influence [5].

Presently, the primary treatment of clubfoot – the Ponseti method – has a 90% rate of effectiveness [6]. According to a survey developed by the Pediatric Orthopedic Society of North America (POSNA), 96.7% of all the orthopedic surgeons who participated in the survey reported that they are currently using Ponseti’s method for idiopathic and non-idiopathic clubfeet [7].

The straightforward and easy access to clubfoot treatment services has great public health implications. Based on recent research, the coverage for clubfoot treatment services in 2015 was less than 25% in low- and middle-income countries, and only 31 countries reported national programs for clubfeet, which were mainly supported by a public-private partnership [8]. To contribute to increasing patients’ access to high quality diagnostic and treatment services, research shows that certain areas must be explored [9–10].

### I. Early clubfoot diagnosis

Early clubfoot diagnosis is one of the most important aspects for families who have children born with this malformation. Research demonstrated that clubfoot should be treated as soon as possible to come up with the most reliable outcomes. To meet these results, a program developed in Uganda by the Ministry of Health emphasized two main strategies: early screening for the deformity and better assessment of the Ponseti method. Moreover, parents should be referred to healthcare professionals in the first week or two [11].

### II. Engaging families in care

Parents play an important role in the proper adherence to the treatment of children born with clubfoot [11]. During the casting phase, 79% of parents did not know that they would have constant responsibility to ensure that their child wore the Ponseti brace correctly. Results highlight that there is an urgent need to improve the education of families about the treatment process and their overall role in it; therefore, devoting sufficient time early on to educate and inform family members is needed [12].

### III. Addressing the barriers and facilitators to access

The barriers identified to the timely presentation of families to specialized healthcare providers for clubfoot treatment include lack of access to medical care and specialized orthopedic physicians, limited public awareness, and lack of information of health units where the Ponseti treatment is available [13]. Thus, a parent advisor should be assigned to each family who has a child born with clubfeet. These initiatives could facilitate better access to medical services and consistency in seeking medical advice [10].

### IV. Mobilization of community in addressing clubfoot related problems

Globally, there is a lack of public knowledge regarding clubfoot which can be associated with few awareness campaigns. It is recommended to increase awareness regarding clubfoot through social media platforms and public campaigns in key locations, such as neonatology clinics. It has been emphasized that this aspect is vital because early management of clubfoot is less invasive [14]. Moreover, working with the community members to raise awareness and provide follow-up throughout patients’ organizations is highly recommended [10].

### V. Psychological support for patients and their families

The medical condition and the treatment regime have a negative impact on the social environment of both the child and the parents. Several indicators, such as negative emotions and perceptions associated with the diagnosis of congenital clubfoot and parents’ concerns about the child’s future, have been assessed throughout recent research studies [15]. There is a demanding need for a regular psychotherapist-delivered counseling session for patients and parents [16].

Currently, little evidence is available in Romania to serve both the proper development of orthopedics management strategies for children born with clubfoot and for the use of the Ponseti method [3, 17]. Currently, the little clubfoot-related research conducted nationally has a strong quantitative focus. To our knowledge, our study is the first qualitative research conducted nationally focusing on patients’ access to clubfoot treatment services, taking into consideration both patients’ and healthcare professionals’ perspectives [5].

Dedicated orthopedics’ health services are severely underfunded. Therefore, training orthopedic surgeons accordingly over a longer period, funding national clubfoot programs, and developing clubfoot-related research are urgent matters that must be tackled. Against this backdrop, our study aimed at assessing the current barriers and facilitators patients are experiencing when attempting to access clubfoot treatment services in Romania.

The main objectives of the study are: (1) to provide an overview of the facilitators and barriers that influence patients to access medical services for the treatment of clubfoot in Romania, and (2) to assess the impact of care-related factors on patients’ well-being (early diagnosis, engaging the families in care, addressing the barriers and the facilitators to access healthcare services, mobilizing the community in addressing clubfoot related problems, and determining the mental health support provided for the patients and their parents).

## Material and Methods

The study has a cross-sectional design, using qualitative methods. In this study, we conducted in-depth individual interviews with the parents of the children and the key medical professionals in the field of pediatric orthopedics.

The target population of the study consisted of parents of children born with clubfeet in Romania and medical personnel across our country. The inclusion criteria for the parents consisted of previous orthopedic medical diagnoses for their children and current residence in Romania. The inclusion criteria for healthcare professionals consisted of current or previous involvement in clubfoot treatment to better assess if the overall diagnosis and treatment were performed accordingly with the recommended methods.

Subject sampling was fulfilled by the snowball technique [18]. We started from a group of known subjects – parents of the children diagnosed with idiopathic clubfeet across Romania – and we built up the sample size using the recommendations made by orthopedic surgeons. The same method was used for healthcare professionals.

Considering that the entire study focused on the five main elements previously-mentioned, the interview for the parents consisted of (1) socio-demographic and introductive questions, (2) the personal choices of the patient’s parents, (3) involvement of families in the proper treatment of clubfeet, (4) monitoring of children diagnosed with clubfoot in the parent/caregiver’s community, (5) mental health support for both the parents and the children, (6) main barriers and experiences encountered by the parents of the children in accessing orthopedic medical services for the treatment of clubfeet nationally, (7) future directions and recommendations. The major constructs of the interview guide developed for key medical personnel are tackling the same main topics, with great emphasis on evidencing the current barriers and facilitators in accessing healthcare services.

Data were analyzed with the Atlas.ti software [19]. Thematic analysis was used to better explore the objectives of the study. The structure of the thematic analysis was composed of themes formed by codes. Additionally, a codebook was redeveloped that had an inductively perspective based on the results of the in-depth interviews and the main variables of interest (early diagnosis of clubfeet, engaging families in care, addressing the barriers and the facilitators to access, mobilizing the community in addressing clubfoot related problems, and analyzing the mental health support provided for the patients and their parents) [20].

## Results

### The 1^st^ group of participants (parents)

Twelve mothers of children between two and 16 years old (six girls and six boys) were interviewed. The study comprises different residence locations: Cluj County, Bihor County, Maramureș County, Hunedoara County, Bacău County, Bistrița-Năsăud County, and Sălaj County. Seven of the children included in the study were diagnosed with unilateral clubfoot, and five were diagnosed with bilateral club feet.

The research analysis uncovered five key themes. These were: early diagnosis, engaging families in the care, addressing the barriers and the facilitators to access, mobilizing the community in addressing clubfoot-related problems, and providing psychological support.

#### Early diagnostic

Only two children were (n=2) diagnosed during the pregnancy scans performed at regular intervals, and some were diagnosed with other serious conditions such as neurological and neuropsychiatric diagnosis. The majority of the children (n=10) were diagnosed right after birth. In most cases, parents reported that their infants received treatment and medical care during the first weeks of life. On the other hand, the treatment was notably delayed in a few cases because parents were initially referred to orthopedic surgeons who were not specialized in treating clubfeet newborns. Obstacles such as lack of awareness and knowledge in the maternity clinics were mentioned several times by the parents.

#### Engaging families in care

Our study indicates that the parents have been greatly involved in the medical process and considered the Ponseti method as soon as they had their first examination. One of the parents reported that they took off the braces before the due period. Some of the reasons highlighted by the mother were that the child was constantly crying due to pain and insomnia.

#### Addressing the barriers and the facilitators to access

The study emphasizes that the quality and the medical experience within the Rehabilitation Hospital in Cluj-Napoca were admirable, and parents were satisfied with the medical services provided. Parents reported that the orthopedic surgeons within the Rehabilitation Hospital were concise about the treatment, and they presented both the benefits and the risks of the entire medical process. The interaction with the rest of the medical team (nurses and orthopedic technicians) was reported to have had a positive impact on the general well-being of the patients. Only one of the parents (n=1) disclosed that nurses and orthopedic technicians should be more friendly and close with the patients.

Conversely, parents reported unpleasant experiences in cities such as Oradea, Timișoara, Baia-Mare, and Bistrița. Inexperienced and untrained doctors, lack of empathy and medical ethics, and clinics not equipped with appropriate medical supplies were the main elements mentioned. The majority of the orthopedic surgeons across Romania use surgical methods and do not consider the Ponseti method.

Besides incidents of poor medical ethics, the parents reported several barriers in accessing high-quality medical services for the treatment of clubfeet, such as: permanent stress and depression, unfavorited access to specialized healthcare professionals, uninformed gynecologists, neonatologists, and family physicians, a small number of professionals who are using the Ponseti method nationally, and financial problems because the cost of the Ponseti braces was not reimbursed by the National Insurance House. In addition, barriers regarding the Ponseti treatment were reported, such as difficulties regarding daily activities (dressing up, sleep etc) and repeated casting processes (each week, for four to eight weeks).

#### Mobilizing the community in addressing clubfoot related problems

Providing follow-up in the patient’s community through social media platforms, awareness campaigns, and parents’ organizations is crucial in improving the general well-being of infants diagnosed with clubfeet. Results show that none of the parents were part of any clubfoot organizations nationally. Most of the parents highlighted that creating clubfoot organizations may positively impact the general well-being of the children. In their perspective, activities such as better mediatization of the condition, exchange of opinions, moral and mental health support, and well-structured information about the specialized pediatric orthopedics at a country level should be the standpoints of an organization.

#### Psychological support for parents and patients

The majority of the parents reported that neither they nor their children received psychological counseling from a specialized physician. To add more, very few parents (n=2) mentioned that they received mental health support and counseling outside the Rehabilitation Hospital. The parents mentioned that receiving psychological counseling from a medical professional within public or private hospitals would have a positive impact. This initiative could help parents better understand the diagnosis of their infant and help them cope with the imposed barriers.

### The 2^nd^ group of participants (healthcare professionals)

Four healthcare professionals from the Rehabilitation Hospital in Cluj-Napoca were interviewed. They all reside in Cluj-Napoca and perform their day-to-day activity at the Department of Pediatric Orthopedics. Three of them were specialized pediatric orthopedic surgeons, and one of them was an orthopedic technician.

Overall, the research identified identical themes within the parents’ population, with greater emphasis on the barriers encountered by parents in accessing high-quality services in the pediatric orthopedics sector.

Firstly, health professionals reported that clubfeet could be diagnosed during the pregnancy period, but unfortunately, the majority of the parents do not receive the diagnosis until the infant is born. Treatment is often delayed because either the neonatologists or gynecologists do not set the correct diagnosis or no concrete recommendations are made regarding the orthopedic doctors who can treat clubfeet nationally. Another recurring problem mentioned by the healthcare professionals is represented by the uninformed family physicians. According to professionals, family doctors should also be informed about the appropriate treatment for clubfeet-born infants, and suggestions should be communicated immediately.

Secondly, results suggest that barriers such as the low number of orthopedic surgeons who perform the Ponseti method nationally, limited accessibility in time and space, the Ponseti braces are not recognized as medical devices; therefore, they are not settled by the National Insurance House, and timeless misdiagnosis were highlighted. The perspectives of both the parents and the healthcare professionals can be efficiently linked, taking into account that their experiences are complementary.

## Discussion

Patients’ access to high-quality healthcare services may be influenced by different elements that impose difficulties in treatment and recovery practices [9].

According to our findings, access to specialized pediatric orthopedic surgeons who perform the Ponseti method nationally is difficult; due to a lack of specialized pediatric orthopedic surgeons who perform the Ponseti method at a national level, the diagnosis of clubfeet can meet major delays in treatment, which can impose serious consequences on the life of newborns. If the treatment is constantly suspended, surgical methods are the only remaining option [21].

Nevertheless, our results show that prenatal diagnosis through ultrasound is an important element in the overall success rate of the treatment. In this regard, gynecologists and neonatologists must immediately recognize the malformation, and future recommendations should be made hastily. This requires favorable collaboration between the Departments of Gynecology, Neonatology, and Orthopedics within a hospital unit [22].

Engaging families in the treatment process is vital for the infant’s life. Data highlights that even though parents were fully engaged in the overall treatment process, they emphasized that information campaigns are still needed. Our findings also support Harmer & Rhatigan [9] who suggest that education must be provided beforehand the initial contact with the healthcare professionals.

Additionally, addressing the barriers regarding patients’ access to clubfeet treatment services is vitally important. The main barriers encountered by the parents had as a focal point the low number of orthopedic surgeons engaged in the treatment of clubfoot. In this regard, policy options should focus on raising awareness around this issue, and the guidelines which emphasize the recommended treatment of musculoskeletal disorders should represent a standpoint for specialists. Moreover, specific opportunities should be provided for healthcare professionals, which have the main objective to guide the Ponseti method.

Similar results were found in studies developed in Uganda that emphasized mainly the same barriers reported in our study. In this context, we can mention the lack of program resources, low socioeconomic status for some families, and poor prenatal support from healthcare professionals [23]. In addition, parents should have support from the health authorities within the country. The County Health Directorates should support families who have lower socioeconomic status and inform physicians about the recommended treatments currently performed for clubfeet.

Addressing barriers to access is perhaps the most context-specific part of improving the overall healthcare access of both the parents and patients to clubfoot services. Health professionals along with the health authorities from a regional and national level must contain their forces to improve the health status of infants [9].

Providing follow-up in the patient community by trained community health workers or public health professionals showed great results for patients. In this way, parents would be able to receive and provide information about their experiences, and adherence to treatment procedures could be significantly improved. Parents reported that they are embracing the idea of developing a parents’ organization in Cluj-Napoca [9].

Lastly, providing psychological support for both parents and patients within the health unit is vital. Some children previously diagnosed with clubfeet may need psychological support. Similarly, parents who bear the responsibility of having a child diagnosed with clubfoot impose serious psychological problems. Our study identified that very few parents accessed psychological counseling with a specialized professional. Health units across the country should provide high-quality psychological services [15].

The straightforward access to clubfoot treatment services in Romania has great public health implications. Research suggests that success was achieved by designing and implementing national programs aimed at improving patients’ access to pediatric orthopedic services. Furthermore, it was demonstrated that it is possible to initiate national programs for clubfoot in a wide range of local conditions using standardized treatment procedures [3].

This study has several limitations. Data obtained throughout the interviews is entirely based on the personal experiences and beliefs of the research subjects, therefore the approach may be subjective and biased. Moreover, the study included a small number of participants, so results cannot be generalized at a national level.

## Conclusion

This study provides insights regarding the current barriers and facilitators in accessing clubfoot treatment services in Romania. The findings suggest that specific training and opportunities should be developed and created within the country to maximize the health workforce in the pediatric orthopedics field. Moreover, globally accepted guidelines for the treatment of clubfoot should represent the essential tool for all surgeons specializing in pediatric orthopedics.

## Acknowledgements

### Conflict of interest

The authors declare no conflict of interest.

### Ethics approval

The study was approved by the Institutional Review Board (or Ethics Committee) of the Department of Public Health (IRB-PH Protocol #2020-201204-006).

### Consent to participate

Verbal consent was obtained from the participants. Due to the COVID-19 pandemic, the study took place entirely over the phone; therefore, information from the informed consents was explained to each participant before the interview began. Both the parents and the healthcare professionals agreed on giving personal information details about the clubfoot patients included in the study, and they agreed for the data to be used for scientific purposes.

### Data availability

Further data is available from the corresponding author on reasonable request.

### Personal thanks

The authors acknowledge Dr. Dan Cosma and Dr. Andrei Corbu from the Iuliu Hatieganu University of Medicine and Pharmacy in Cluj-Napoca and Carol Davila University of Medicine and Pharmacy in Bucharest, who helped us contact the participants. Moreover, we would like to express our sincere appreciation to Ms. Monica-Georgiana Brînzac from the Department of Public Health, Faculty of Political, Administrative and Communication Sciences, Babes-Bolyai University, who helped us revise an early draft of the paper.

### Authorship

BOD and MIU designed the study and analytical approach. BOD and MIU conceived and designed the analysis. BOD performed the data analysis and interpreted the results. BOD wrote the first draft of the manuscript. BOD and MIU critically revised the manuscript and approved the final version.
